# Alcohol assessment & feedback by e-mail for university student hazardous and harmful drinkers: study protocol for the AMADEUS-2 randomised controlled trial

**DOI:** 10.1186/1471-2458-13-949

**Published:** 2013-10-10

**Authors:** Jim McCambridge, Marcus Bendtsen, Nadine Karlsson, Ian R White, Preben Bendtsen

**Affiliations:** 1 Faculty of Public Health & Policy, London School of Hygiene and Tropical Medicine, 15-17 Tavistock Place, WC1H 9SH London, UK, England; 2 Department of Medicine and Health, Linköping University, 581 83 Linköping, Sweden; 3 Department of Computer and Information Science, Linköping University, Linköping, 581 83, Sweden; 4 MRC Biostatistics Unit, Institute of Public Health, Robinson Way, CB2 0SR Cambridge, UK, England

## Abstract

**Background:**

Alcohol is responsible for a large and growing proportion of the global burden of disease, as well as being the cause of social problems. Brief interventions are one component of comprehensive policy measures necessary to reduce these harms. Brief interventions increasingly take advantage of the Internet to reach large numbers of high risk groups such as students. The research literature on the efficacy and effectiveness of online interventions is developing rapidly. Although many studies show benefits in the form of reduced consumption, other intervention studies show no effects, for reasons that are unclear. Sweden became the first country in the world to implement a national system in which all university students are offered a brief online intervention via an e-mail.

**Methods/Design:**

This randomized controlled trial (RCT) aims to evaluate the effectiveness of this national system comprising a brief online intervention among university students who are hazardous and harmful drinkers. This study employs a conventional RCT design in which screening to determine eligibility precedes random allocation to immediate or delayed access to online intervention. The online intervention evaluated comprises three main components; assessment, normative feedback and advice on reducing drinking. Screening is confined to a single question in order to minimise assessment reactivity and to prevent contamination. Outcomes will be evaluated after 2 months, with total weekly alcohol consumption being the primary outcome measure. Invitations to participate are provided by e-mail to approximately 55,000 students in 9 Swedish universities.

**Discussion:**

This RCT evaluates routine service provision in Swedish universities via a delay in offer of intervention to the control group. It evaluates effects in the key population for whom this intervention has been designed. Study findings will inform the further development of the national service provision.

**Trial registration:**

ISRCTN02335307.

## Background

Alcohol is responsible for a large and growing proportion of the global burden of disease, as well as being the cause of social problems. In 2010 it was estimated to cause 5.5% of the total burden and approximately 5 million deaths globally in that year, an increase of approximately one third since 1990 [1]. Existing evidence suggests the optimal strategy for reducing societal alcohol problems is to combine population-level interventions that seek to influence the price, availability and cultural acceptability of hazardous and harmful drinking in concert with individual-level brief interventions delivered in health systems and elsewhere [2,3]. This combination should be expected to produce multi-level benefits, and the dissemination of brief interventions has a key role to play in enhancing public understanding of the nature of alcohol problems, which is very weak. Face-to-face brief interventions are typically offered opportunistically by non-specialists in routine contacts with patients attending health care services and take only a few minutes to deliver [2,4].

Evidence for the efficacy of brief interventions is based on randomized controlled trials and systematic reviews, which have consistently identified small effects on drinking behaviour [5-8]. National programmes have been slow to be implemented as there have been persistent doubts about the strength of this evidence, and the potential contribution to public health. Not unrelatedly, there have been longstanding difficulties in persuading generic health and welfare practitioners to embrace this work in routine practice [9,10]. In the era of the internet there are now other ways to reach large numbers of hazardous and harmful drinkers which overcome implementation problems due to practitioner reluctance to discuss drinking [9].

Online interventions may be more cost-effective than face-to-face interventions. The expectation is that they are both less costly and less effective than face-to face interventions [11]. It also seems likely that face-to-face interventions may be necessary for those with more severe problems and difficulties in changing behaviours and that it will be necessary to develop intervention programmes that combine both elements. Internet interventions may also be more acceptable to those targeted, though this will not be important to public health unless they can also be demonstrated to be effective and cost-effective [12,13].

The research literature on the efficacy and effectiveness of online interventions is developing rapidly. Systematic reviews provide preliminary evidence of efficacy for a range of computerised interventions [11,14,15], though there are also examples of apparently well designed interventions (e.g. [16]) not being found to be effective [17]. So, although many studies show benefits in the form of reduced consumption, other intervention studies show no effects, for reasons that are unclear [18,19].

Many existing computerised brief intervention studies target university student populations [15]. Heavy drinking among university students is a global phenomenon [20-22], and is particularly well established in countries with cultures and traditions of heavy drinking. There are now effectiveness reviews of normative feedback and similar interventions delivered face to face and online among students [11,23,24]. Many previous studies have, however, evaluated computerised rather than online interventions, requiring participants to attend laboratories or similar settings [14,15,19].

Kypri and colleagues randomised 2,435 hazardous and harmful drinkers in one Australian university in the THRIVE trial evaluating an online intervention [25]. This intervention achieved a 17% reduction in alcohol consumption in an online assessment and feedback group compared to a non-intervention assessment-only group, which subsequently attenuated to an 11% difference after 6 months [25]. Kypri and colleagues also undertook the eSBINZ trials in 7 universities in New Zealand among Maori and non-Maori students respectively [12]. Similar effects to that seen in THRIVE were obtained among Maori students [26], though effects among non-Maori students were smaller.

Swedish students drink alcohol heavily [21,27] and Swedish universities accept a duty of care in relation to drinking among their students, which has obvious consequences for the fulfilment of their educational function within society. An online intervention originally developed by the Lifestyle Intervention Research group at Linköping University [28] has now been adopted as the basis for a national system. This made Sweden the first country in the world to implement any national system devoted to addressing student drinking. This intervention is based upon an initial e-mail to students from the student healthcare service, providing a link to a website for assessment and feedback. The core content involves assessment, feedback on recommended limits of alcohol consumption and normative comparisons of drinking with Swedish students of the same age and sex and tailored advice.

The first trial of this intervention found no differences between brief and more extensive normative feedback content though attrition problematically reduced available sample size in a feasibility study [29]. We then undertook a further trial as a large pilot study with outcome data provided by 2,400 students [30]. This pilot study successfully employed incentives to enhance follow-up rates on the previous trial, though attrition was highly differential between-groups, with the non-intervention control group approximately 10% more likely to participate, not having previously received an e-mail relating to alcohol in contrast to two other randomised groups [30]. These data informed the design of the AMADEUS-1 trial, a study with a range of unconventional characteristics intended to a address various methodological weaknesses and unresolved design questions in this field [31].

A key issue which has presented difficulties is the determination of the most appropriate control groups, due largely to overlap and similarities between content integral to interventions based on assessment and feedback and research assessments [32,33]. Content similarities and overlap entail contamination, as the control group is exposed to the intervention content whose effects are being evaluated [34], and this applies to relatively brief screening tools, such as the 10-item AUDIT (Alcohol Use Disorders Identification Test) alcohol screening questionnaire [35], as well as lengthier assessments [36]. Brief intervention researchers have long been interested in the possibility that assessment of alcohol consumption per se can reduce drinking [5,33,37]. Observed mean reductions in drinking in non-intervention control groups in brief intervention trials are approximately 20% at later follow-ups [38,39]. A systematic review of randomized evaluations of assessment reactivity in brief intervention studies found evidence of small effects [40]. When attention was restricted to university student populations, however, somewhat more consistent and stronger effects were apparent [40]. If simply by answering questions on one’s drinking, however, does subsequently lead to reduced drinking, large-scale implementation of simple screening surveys as interventions among university students might have a considerable public health potential [28,33].

These considerations led to the AMADEUS-1 trial. This study randomised e-mail addresses and employed a no contact control group, without any screening or selection of participants according to alcohol risk status. All participants were blind to trial participation at all stages of the study. Outcome evaluation was via a seemingly unrelated 15- item cross-sectional lifestyle survey containing trial outcomes derived from 3 items [31]. The use of deception in this study requires justification and we offer this elsewhere [41]. In a universal prevention approach, some evidence of benefit was found for an assessment only group, with no additional benefit of feedback (though there was some per protocol evidence for this) [42]. This study adds to existing evidence of problems with screening and assessing control groups in conventional trial designs in this area. With this design the AMADEUS-1 trial was unable to provide an intention-to-treat evaluation of possible effects among hazardous and harmful drinkers, the key target group, and was reliant on a per-protocol analysis for determination of effects in this group. AMADEUS-2 thus aims to provide an evaluation of online assessment and feedback intervention effects among hazardous and harmful drinkers among the Swedish university student population.

## Methods/Design

This is a two-arm parallel groups trial in which routine provision of online assessment and feedback intervention (Group 1) is compared with non-intervention (Group 2) by delaying online access to intervention. In routine practice the timing of intervention delivery varies across Sweden and we take advantage of this lack of standardisation of timing to implement random allocation in this effectiveness evaluation study. This conventional trial design is informed by previous reactivity findings and identifies hazardous and harmful drinkers by means of a single screening question. Unlike the AMADEUS-1 trial, there is no blinding in this study. The study was approved by the Regional Ethical Committee in Linköping, Sweden (Number: 2013/46-31).

### Participants & setting

The study is undertaken in 9 of the 26 student health care centres, each providing services to one university or college in Sweden. These institutions have been selected on the basis that they have not previously been involved in randomised controlled trials in our research programme. All students at the 9 colleges during the spring term (i.e. in terms 2, 4 and 6) were sent an e-mail inviting them to answer a single question about their drinking, and if eligible for trial participation, were provided with information permitting informed consent. The single question is the third item of the AUDIT questionnaire on the frequency of heavy episodic drinking [43]. Standalone single alcohol screening questions have been validated as identifying hazardous and harmful drinkers in different settings [44,45] and this type of drinking is particularly important in this population [21]. Students who were drinking more frequently than once per month 5 drinks [of 12 grams of alcohol] or more for men or 4 drinks or more for women are deemed eligible for trial participation. This approach was also used by Walters and colleagues [46] who were similarly concerned to avoid reactivity to screening.

### Recruitment, randomisation and other study procedures

The initial e-mail is sent from the participating student health care services as usual, to approximately 54,000 e-mail addresses in total. The usual introductory text is altered so as to invite study participation. After screening positive and receiving study information, participants clicked on a button to provide informed consent and are immediately randomised to intervention or control conditions. The former group gain immediate access to the intervention and the latter group are advised that they will be able to access the intervention in two months. Two months later both groups are sent an identical e-mail by us. This reminds participants of the study and invites them to undertake the study follow-up to access the intervention. There are a total of four reminders (making five opportunities to respond in all), initially at weekly intervals, then at shorter intervals, with the final e-mail making clear that this is the last opportunity to respond, and allowing 2 days to do so. There are no incentives used to encourage study participation or retention.

Each participant is allocated numbers 1 or 2 following a uniform distribution. This is done using Java’s built in random number generator (java.util.Random). Randomisation is thus fully computerized, does not employ any strata or blocks, and is not possible to subvert, as this and all subsequent study processes are fully automated.

### Intervention content

The intervention group receives feedback immediately upon completion of the assessment consisting of three statements summarizing their weekly consumption, their frequency of heavy episodic drinking and their highest blood alcohol concentration during the last four weeks, comparing drinking patterns against the safe drinking limits established by the Swedish Institute for Public Health [29]. After this follows comprehensive normative feedback with information describing participants’ alcohol use compared to their peers in Swedish universities, and, if applicable, personalized advice concerning the importance of reducing any unhealthy levels or pattern of consumption. The feedback can be printed out by the student. A demonstration version of the assessment and feedback intervention can be viewed at http://demo.livsstilstest.nu.

### Sample size

The marginal costs involved in delivering online interventions to large numbers of participants in both routine practice and in R & D studies are low, and much lower than other brief interventions, after the developmental costs are met [47,48]. Therefore even very small effects are likely also to be highly cost effective above the basic threshold cost involved in providing the service. These observations lead us to believe that the sample should be as large as possible, in order to detect very small effects.

To assist study planning we undertook an illustrative power calculation. To detect an effect size of 0.1 standard deviations between the two groups with 5% significance level and 80% power, we required 1,600 individuals analysed per group. Assuming a follow-up rate of 50%, we should aim to recruit 3,200 individuals per group i.e. 6,400 in total. We had no data on the number of screen positives who may be willing to participate in this trial and assumed approxiamtely 70% would do so, meaning that we would need to identify approximately 8,000 hazardous and harmful drinkers. In order to identify these number of participants, e-mails could need be sent to approximately 40,000 students with an average response rate of 40% (i.e. n = 16,000) and a prevalence rate of 60% among responders (giving n = 9,600). We could not be confident of these estimates as, for example, patterns of e-mail address use vary considerably between colleges, being compulsory in some institutions and rarely used in others. We therefore decided to undertake the study in 9 colleges with a total student enrolment of approximately 54,000 students.

### Outcome evaluation

This study uses a single 2 month follow-up interval, after which the control group will gain access to the intervention. The study thus provides information only on the short term effects of the intervention. If we find no short term effects, we will conclude the intervention will not have any long term effects.

The primary outcome is total weekly alcohol consumption. This is computed as the sum of alcohol consumption for each of the 7 days in a typical week. Secondary outcomes are the proportions still drinking above national guidelines [49], frequency of drinking (number of days per week), quantity of drinks per drinking day, frequency of heavy episodic drinking as defined in the screening question, highest estimated blood alcohol concentration (eBAC) and stage of change.

### Statistical methods

As there is no research assessment at study entry, information on the sample at this point is restricted to university, term, time from sending of invitation email to consent, and (from the screening question) the frequency of heavy episodic drinking. At follow-up, we will have available additional information that is not possible, or not likely, to have been altered or altered differentially during the study period and which will therefore be treated as baseline information: age, gender, faculty, marital status and language used (Swedish or English). We also use measures of engagement with the study (mode of data collection, number of follow-up emails sent and elapsed time before follow-up was completed).

All outcomes will be compared between randomised groups under the intention-to-treat principle (that is, including all randomised individuals in their originally randomised groups). Continuous outcome measures will be assessed for skewness. If not skewed, they will be analysed by linear regression with results reported as a mean reduction. If skewed, they will either be log-transformed and analysed by linear regression [17], or analysed by negative binomial regression [25], and results will be reported as% reduction. Drinking above national guidelines will be analysed by logistic regression and reported as a% reduction in odds. Frequency of HED occasions will be analysed by ordered logistic regression and reported as a% reduction in odds for exceeding any level. All regression analyses will be first unadjusted and then adjusted for frequency of heavy episodic drinking at baseline, age, university, and gender, using the first two as continuous variables; the adjusted analysis will be primary.

Missing outcome data will be initially handled by a complete-cases analysis, which assumes that the data are missing at random. The plausibility of this analysis will be assessed by exploring the trend in outcomes across increasing numbers of follow-up emails, and a sensitivity analysis will use the repeated attempts model [50] which allows data to be missing not at random on the alternative assumption that the association between outcome and response is the same across follow-up emails.

Effect modification tests for frequency of heavy episodic drinking at baseline, age, university, and gender will be undertaken for the primary outcome only, using the first two as continuous variables.

## Discussion

This RCT evaluates routine service provision in Swedish universities via a delay in offer of intervention to the control group, in common with other studies in the AMADEUS research programme. This delay raises ethical issues, though in this study there is no deception used as there was in the AMADEUS-1 trial [41], and the ethical issues associated with the delay are not viewed as challenging. This study evaluates effects in the key population for whom this intervention has been designed. Study findings will inform the further development of the national service provision. There are many well known reasons to identify and seize opportunities to undertake randomised studies as they provide more rigorous estimates of intervention effects than non-randomised studies. We suggest that they may be under-utilised in evaluations of existing services for which they can be useful in guiding further developments.

## Abbreviations

AUDIT: Alcohol use disorders identification test.

## Competing interests

PB and MB own a company that has developed the online intervention evaluated in this study and that develops and distributes computerised lifestyle interventions. Other authors have no relationships with any companies that might have an interest in the submitted work; our spouses, partners, or children have no financial relationships that may be relevant to the submitted work; and we have no non-financial interests that may be relevant to the submitted work.

## Authors’ contributions

JM and PB had the original idea for the study, obtained funding and led on its design. PB has overall responsibility for study implementation. MB does all computer programming associated both with interventions delivery and study data collection. IR led on statistical aspects of this study, with inputs from NK and JM. JM wrote the first draft of the study protocol to which all authors contributed. All authors read and approved the final manuscript.

## Pre-publication history

The pre-publication history for this paper can be accessed here:

http://www.biomedcentral.com/1471-2458/13/949/prepub
